# The N-recognin UBR4 of the N-end rule pathway is required for neurogenesis and homeostasis of cell surface proteins

**DOI:** 10.1371/journal.pone.0202260

**Published:** 2018-08-29

**Authors:** Sung Tae Kim, Yoon Jee Lee, Takafumi Tasaki, Joonsung Hwang, Min Jueng Kang, Eugene C. Yi, Bo Yeon Kim, Yong Tae Kwon

**Affiliations:** 1 Protein Metabolism Medical Research Center and Department of Biomedical Sciences, College of Medicine, Seoul National University, Seoul, Republic of Korea; 2 Center for Pharmacogenetics and Department of Pharmaceutical Sciences, School of Pharmacy, University of Pittsburgh, Pittsburgh, PA, United States of America; 3 Medical Research Institute, Kanazawa Medical University, Ishikawa, Japan; 4 World Class Institute, Anticancer Agents Research Center, Korea Research Institute of Bioscience and Biotechnology, Ochang, Cheongwon, Republic of Korea; 5 Department of Molecular Medicine and Biopharmaceutical Sciences, School of Convergence Science and Technology and College of Medicine or College of Pharmacy, Seoul National University, Seoul, Republic of Korea; 6 Ischemic/Hypoxic Disease Institute, College of Medicine, Seoul National University, Seoul, Republic of Korea; CHA University, REPUBLIC OF KOREA

## Abstract

The N-end rule pathway is a proteolytic system in which single N-terminal amino acids of proteins act as a class of degrons (N-degrons) that determine the half-lives of proteins. We have previously identified a family of mammals N-recognins (termed UBR1, UBR2, UBR4/p600, and UBR5/EDD) whose conserved UBR boxes bind N-degrons to facilitate substrate ubiquitination and proteasomal degradation via the ubiquitin-proteasome system (UPS). Amongst these N-recognins, UBR1 and UBR2 mediate ubiquitination and proteolysis of short-lived regulators and misfolded proteins. Here, we characterized the null phenotypes of UBR4-deficient mice in which the UBR box of *UBR4* was deleted. We show that the mutant mice die around embryonic days 9.5–10.5 (E9.5–E10.5) associated with abnormalities in various developmental processes such as neurogenesis and cardiovascular development. These developmental defects are significantly attributed to the inability to maintain cell integrity and adhesion, which significantly correlates to the severity of null phenotypes. UBR4-loss induces the depletion of many, but not all, proteins from the plasma membrane, suggesting that UBR4 is involved in proteome-wide turnover of cell surface proteins. Indeed, UBR4 is associated with and required to generate the multivesicular body (MVB) which transiently store endocytosed cell surface proteins before their targeting to autophagosomes and subsequently lysosomes. Our results suggest that the N-recognin UBR4 plays a role in the homeostasis of cell surface proteins and, thus, cell adhesion and integrity.

## Introduction

The N-end rule pathway is a proteolytic system in which single N-terminal amino acids act as degradation determinants, called N-degrons [1–3]. Known N-degrons include Arg, Lys, His (type-1, positively charged), Trp, Phe, Tyr, Leu, and Ile (type-2, bulky hydrophobic) exposed at the N-termini of proteins in humans [4, 5]. These N-terminal residues are selectively recognized by recognition components, called N-recognins [6]. The protein substrates carrying N-degrons can be degraded by either the UPS or the autophagy-lysosome system (hereafter autophagy) [7–9]. In the UPS, N-recognins induce ubiquitination and proteasomal degradation of the substrates [10, 11]. The mammalian genome encodes a family of N-recognins (UBR1, UBR2, UBR4/p600, and UBR5) that recognize type-1 N-degrons through their conserved UBR boxes [12, 13]. Amongst these N-recognins, UBR1 and UBR2 with a size of 200 kDa are single polypeptide E3 ligases that bind all type-1 and type-2 N-degrons [4, 14]. These RING finger E3 ligases mediate ubiquitination of short-lived regulators in the cytosol and nucleus as well as misfolded proteins [7, 15, 16]. UBR4 is a 570-kDa protein that binds both type-1 and type-2 N-degrons [6, 17, 18]. This poorly characterized N-recognin does not have a known ubiquitination domain but is required for optimal degradation of a model N-end rule substrate as well as ubiquitination of huntingtin (HTT) proteins such as 73 poly-glutamine repeat-bearing mutant HTT (73Q-HTT) and 175Q-HTT [19]. UBR4 and UBR5 are key regulators that synthesize K11/K48-branched heterotypic ubiquitin chains, which are induced and destined for proteasomal degradation during proteotoxic stress [19]. UBR5 is a 300 kDa E3 ligase that preferentially binds type-1 N-degrons [6, 20, 21]. The HECT domain protein mediates ubiquitination of short-lived proteins such as ATMIN [22]. Besides the known N-recognins, the mammalian genome encodes at least three more UBR box proteins, UBR3, UBR6, and UBR7 [6, 23], whose functions remain largely unknown [24–26]. In addition to the UPS, N-degrons can induce proteolysis via autophagy. In the autophagic N-end rule pathway, the autophagic adaptor p62/SQSTM1/Sequestosome-1 binds type-1 and type-2 residues and deliver the substrates to autophagosomes, leading to lysosomal proteolysis [27, 28]. The N-end rule substrates of p62 include N-terminally arginylated proteins such as molecular chaperones that reside in the endoplasmic reticulum (ER) and a number of cytosolic proteins [8, 29].

Human UBR4 has been identified as a microtubule-associated protein [6, 30–32]. Subsequent studies by us and others have shown that UBR4 facilitates ubiquitination and proteasomal degradation of short-lived proteins, including N-end rule substrates [6] as well as mutant huntingtins [19]. Additionally, UBR4 promotes ubiquitination of ATP-citrate lyase (ACLY), a key regulator of fatty acid biogenesis, suggesting that UBR4 has a role in tumor progression and lipid synthesis [33]. UBR4 has also been implicated in a number of seemingly random processes outside the N-end rule pathway, ranging from the pathogenesis of neurodegeneration [34, 35] to the extracellular secretion of microvesicles and ectosomes [36, 37]. Other studies showed that UBR4 is required for cellular immortalization and transformation induced by human papillomavirus (HPV) E7 [38–41], which can be in part attributed to its role in cell-to-cell adhesion and integrin-induced apoptosis [18]. In *Arabidopsis*, UBR4/BIG has been shown to mediate the vesicular transport of auxin, a master hormone [42]. Despite these isolated observations, the general role of UBR4 at the cellular level and developmental stages remains unclear.

We have previously constructed UBR4-knockout mice in which a UBR box-containing region, spanning exons 36 through 42, was replaced with IRES (internal ribosome entry site)-translated tau-lacZ [32]. The mutant mice die during embryogenesis from E9.5 through E10.5. UBR4-deficient mouse embryos exhibit vascular defects in the yolk sac (YS) [31, 32]. The development of blood vessels in the *UBR4*^*-/-*^ YS advances via vasculogenesis but is arrested during angiogenic remodeling of primary capillary plexus [32]. In the YS, UBR4 marks endoderm-originated, autophagy-specialized cells that support angiogenic remodeling of mesoderm-derived vascular cells and supply autophagy-produced amino acids during early embryogenesis [31, 32]. In cultured cells, UBR4 is a substrate of autophagy, and UBR4 loss results in the induction of autophagy and its flux, implicating UBR4 in autophagy modulation [32]. UBR4-deficient mice were also generated by others [43, 44]. UBR4 knockout mice, in which exon 1 was deleted, died at E11.5-E13.5 associated with defects in embryonic and placental development [43]. When exon 1 was deleted in mice by mating with transgenic mice expressing Cre driven by Sox2 promoter, embryonic growth was arrested leading to death around E12.5-E14.5 with defects in cardiogenesis [43, 44].

During the development of the nervous system, cell-cell adhesion regulates the differentiation, proliferation, and migration of neural stem/progenitor cells [45, 46]. Cell adhesion is mediated by membrane-associated proteins such as cadherins, by which cells bind the extracellular matrix or interact with other cells. Of classical cadherins, neural or N-cadherin (Cadherin-2), enriched in the neural system, is a component of adherens junction, in which it interacts with delta-catenin (p120), beta-catenin, and gamma-catenin [46, 47]. N-cadherin is also broadly expressed in non-neuronal tissues such as the cardiac system [46, 48]. Mice lacking N-cadherin exhibit embryonic lethality at E10 due to impaired cell adhesion in the primitive heart and YS [46, 48]. Similar developmental defects in cell adhesion are observed in mice lacking other adhesion proteins that interact with N-cadherin [46].

In this study, we further characterized the null phenotypes of UBR4-deficient mouse embryos. Our results show that *UBR4*^*-/-*^ embryos develop multiple developmental defects including neurogenesis. Various types of cells in UBR4-deficient embryos lose directionality and are morphologically abnormal, which is at least in part attributed to the impairment in cell adhesion. UBR4-loss induces the depletion of cell surface proteins associated with the inability to generate multivesicular bodies (MVBs). Our results reveal an essential role of UBR4 in non-proteolytic processes such as the homeostasis of cell surface proteins and, thus, cell adhesion and integrity.

## Results

### Mouse embryos lacking UBR4 die during midgestation associated with multiple developmental abnormalities

**Fig 1:**
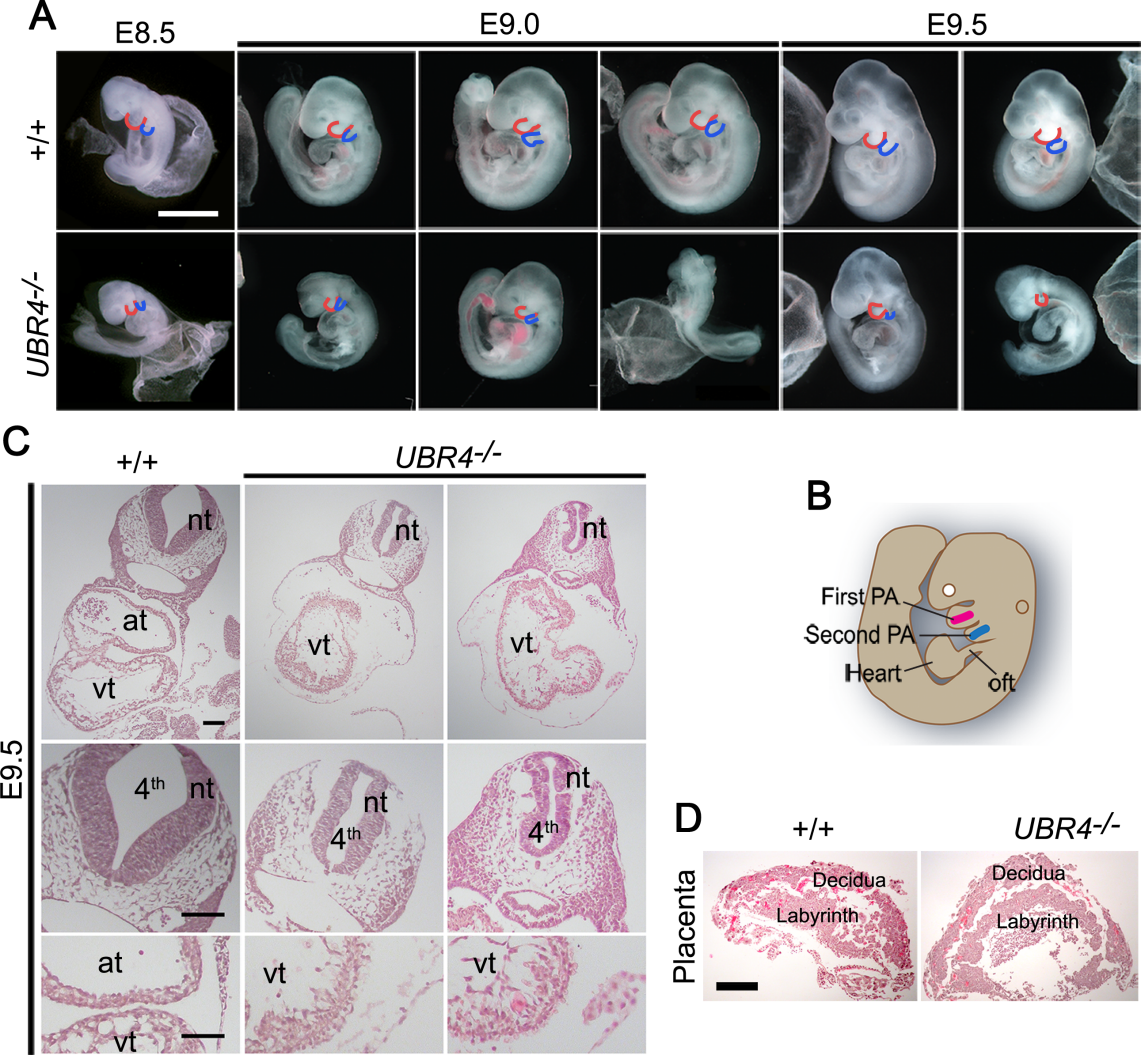
Mouse embryos lacking UBR4 die at midgestation associated with multiple developmental abnormalities. (**A**) Gross morphology of +/+ and *UBR4*^*-/-*^ mouse littermates at E8.5-E9.5. UBR4 mutants exhibit embryonic growth retardation and malformed pharyngeal arches. The red and blue lines represent the first PA and second PA, respectively (scale bar = 1 mm). (**B**) Anatomical illustration of the pharyngeal arch (PA) and heart of the mouse embryo at E9.0. First PA mesodermal cells (red) move to the developing heart tube for the right ventricular myocardium at E7.5–8.0, while second PA mesodermal cells (blue) migrate to the outflow tract (oft) of the myocardium at around E9.5-E10. (**C**) Hematoxylin and eosin (H&E) staining on cross sections of +/+ and *UBR4*^*-/-*^ embryos at E9.5. nt, neural tube; at, atrium; vt, ventricles; 4^th^, fourth ventricle (scale bar = 100 μm). (**D**) H&E staining of the placenta of +/+ and *UBR4*^*-/-*^ mouse littermates at E9.5 (scale bar = 500 μm).

We have previously constructed UBR4 knockout mice, in which a 4.1 kb-DNA fragment encoding the UBR box was deleted [31, 32]. The mutant mouse embryos died at midgestation (~E9.5–10.5) associated with vascular defects in the YS [31, 32]. In this study, we characterized the null phenotypes of the mutant embryos proper. When gross phenotypes were examined, *UBR4*^*-/-*^ embryos developed normally until E8.0 but began to show growth retardation at E8.5, resulting in developmental arrest at E9.5 (Fig 1A). By E10.5, the majority of homozygous mutants were in the course of dying or found dead. No live mutants were retrieved at and beyond E11.5. These results suggest that UBR4 is indispensable for embryonic development at midgestation.

One distinct developmental defect was that the majority of *UBR4*^*-/-*^ embryos between E8.5 and E9.5 had smaller and malformed pharyngeal arches as compared to control embryos (Fig 1A). The abnormalities were more prominent in the second pharyngeal arch, which is derived from pharyngeal epithelium and mesenchymal cells (Fig 1A and 1B, blue line). It is known that mesoderm cells in the first arch migrate to the developing heart tube, contributing to right ventricular myocardium at E7.5-E8.0 (Fig 1B). By E9.5-E10, mesodermal cells in the second arch also migrate to contribute to outflow tract myocardium [49]. Consistent with these developmental fates, *UBR4*^*-/-*^ embryos almost invariably exhibited defects in cardiovascular development characterized by thin myocardium and loosely associated cardiac fibroblasts (Fig 1C). Despite such pleiotropic abnormalities, the placenta of *UBR4*^*-/-*^ embryos at E9.5 did not show severe morphological and histological abnormalities in the spongiotrophoblast and labyrinthine layer (Fig 1D), indicating that these null phenotypes observed in *UBR4*^*-/-*^ embryos are not secondary to the YS vascular defects. These results suggest that UBR4 is required for embryonic development at and beyond E8.0.

### UBR4 is required for embryonic neurogenesis

**Fig 2:**
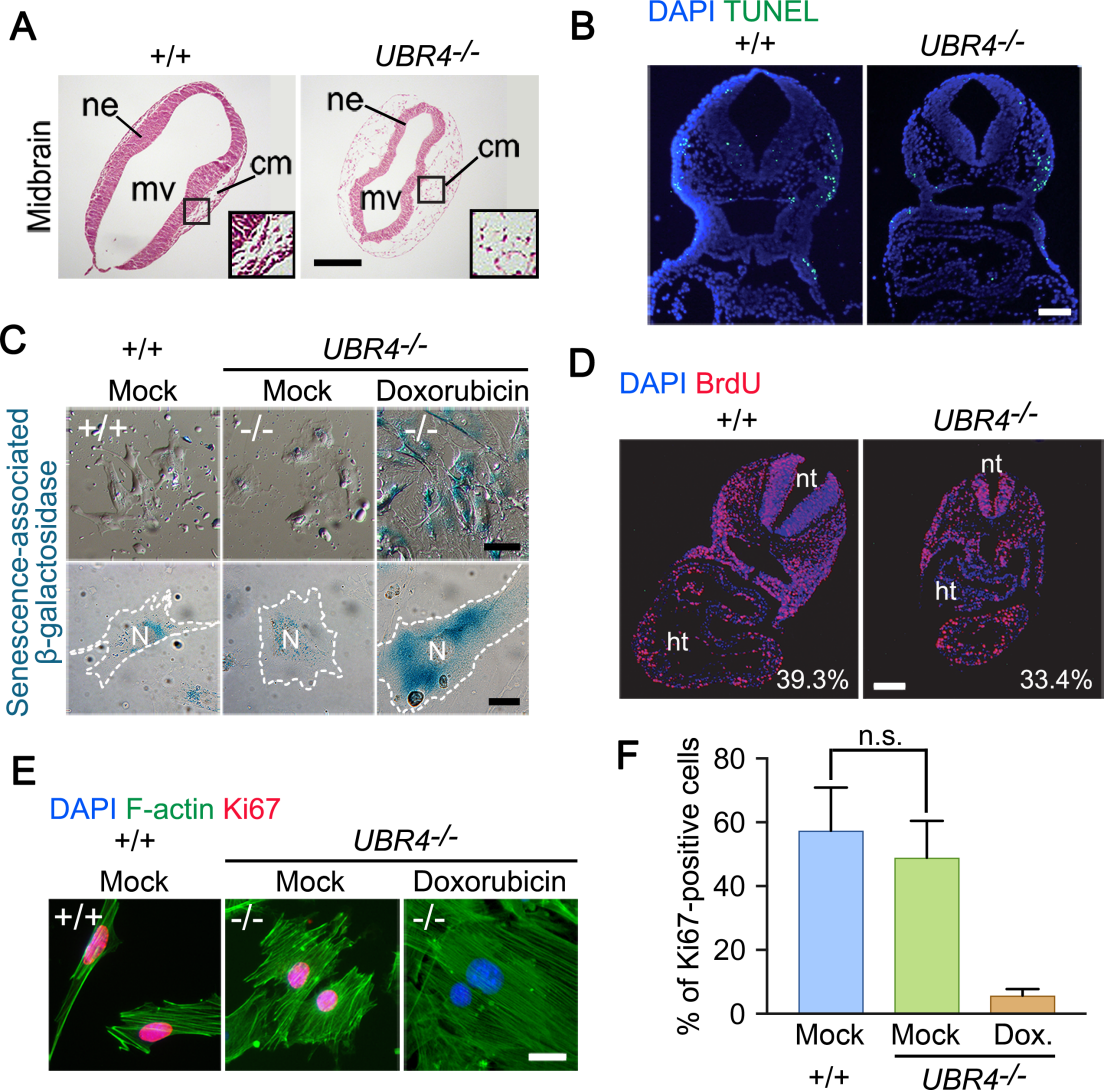
The null phenotypes of *UBR4*^*-/-*^ embryos are not due to cell death or misregulation in cell cycle or proliferation. (**A**) H&E staining of the midbrain of +/+ and *UBR4*^*-/-*^ mouse embryos at E9.5. Insets: magnified image of area enclosed by lined boxes. cm, cephalic mesenchyme tissue; mv, mesencephalic vesicle; ne, neuroepithelium of midbrain region (scale bar = 200 μm). **(B**) TUNEL assay of +/+ and *UBR4*^*-/-*^ embryos at E9.5 (scale bar = 150 μm). (**C**) Cytochemical staining of senescence-associated β-galactosidase (SA-βgal) activity in +/+ and *UBR4*^*-/-*^ MEFs. For a positive control, *UBR4*^*-/-*^ MEF were treated with 50 nM doxorubicin for 7 days. White dotted line delineates the cell boundary (scale bar = 30 and 15 μm, respectively). N, nucleus. (**D**) BrdU incorporation assay of +/+ and *UBR4*^*-/-*^ embryos at E9.5. S-phase indices were determined to be 39.3% and 33.4% for +/+ and *UBR4*^*-/-*^ embryos, respectively. nt, neural tube; ht, heart (scale bar = 150 μm). (**E**) Immunostaining of F-actin (filamentous actin) and Ki67 in +/+ and *UBR4*^*-/-*^ MEFs. For a negative control (G_0_ phage), *UBR4*^*-/-*^ MEF were treated with 50 nM doxorubicin for 7 days. (scale bar = 15 μm). (**F**) Quantitative of Ki67-positive signals in +/+ and *UBR4*^*-/-*^ MEFs. The percentage of Ki67-positive cells among DAPI-positive cells was calculated in 5 randomly selected fields at a magnification of 200x. Statistically no significant difference was noted between +/+ and *UBR4*^*-/-*^ MEFs (n.s., not significant; *p = 0*.*8591*). Data are presented as mean percentage ± SEM. Statistical significance was determined using one-way analysis of variance (ANOVA) and Tukey test as a post hoc comparison.

To further characterize the *in vivo* role of UBR4 in embryonic development, we histologically examined the cross sections of +/+ and *UBR4*^*-/-*^ mouse embryos at E8.5 through E10.5. One prominent histological phenotype was noticed in neurogenesis (Figs 1C and 2A). The neural tube of *UBR4*^*-/-*^ embryos at E9.5 was thinner and rough on the surface of the neuroepithelium as compared with littermate controls (Figs 1C and 2A). The fourth ventricle in *UBR4*^*-/-*^ embryos was morphologically abnormal, shrunken, and almost closed as compared with control embryos (Fig 1C). The areas between mesencephalic ventricles and cephalic mesenchyme tissues were significantly reduced (Fig 2A). The cells located in cephalic mesenchyme tissues were sparsely found and did not show directionality (Fig 2A). These results suggest that UBR4 is required for neurogenesis.

We next determined whether the aforementioned developmental defects are secondary to cell proliferation and death. TUNEL assays on cross sections of +/+ and *UBR4*^*-/-*^ embryos showed that the severity of null phenotypes correlated to the degree of apoptotic induction (Fig 2B). However, the overall levels of apoptotic cells were not significantly influenced by UBR4 knockout. We therefore examined cellular senescence by monitoring the activity of β-galactosidase, an indicator of senescence. *UBR4*^*-/-*^ MEFs did not contain a comparable level of β-galactosidase activity and, thus, had the capacity to undergo cell cycle (Fig 2C). To determine whether UBR4 loss affects cell proliferation, we measured the S-phase index of *UBR4*^*-/-*^ embryos. We injected bromodeoxyuridine (BrdU) into pregnant females, harvested embryos, and examined the incorporation of BrdU into S-phase DNA. Anti-BrdU immunostaining analyses on the cross sections of +/+ and *UBR4*^*-/-*^ embryos at E9.5 showed that the S-phase index of *UBR4*^*-/-*^ embryos was largely comparable to that of +/+ embryos (Fig 2D). Consistently, immunostaining analyses of Ki67, a marker of proliferating cells, showed that *UBR4*^*-/-*^ MEFs were mitotically active as compared to +/+ cells (Fig 2E and 2F). These results suggest that the developmental abnormalities of *UBR4*^*-/-*^ embryos are not subsidiary to reduced cell proliferation or increased cell death.

**Fig 3:**
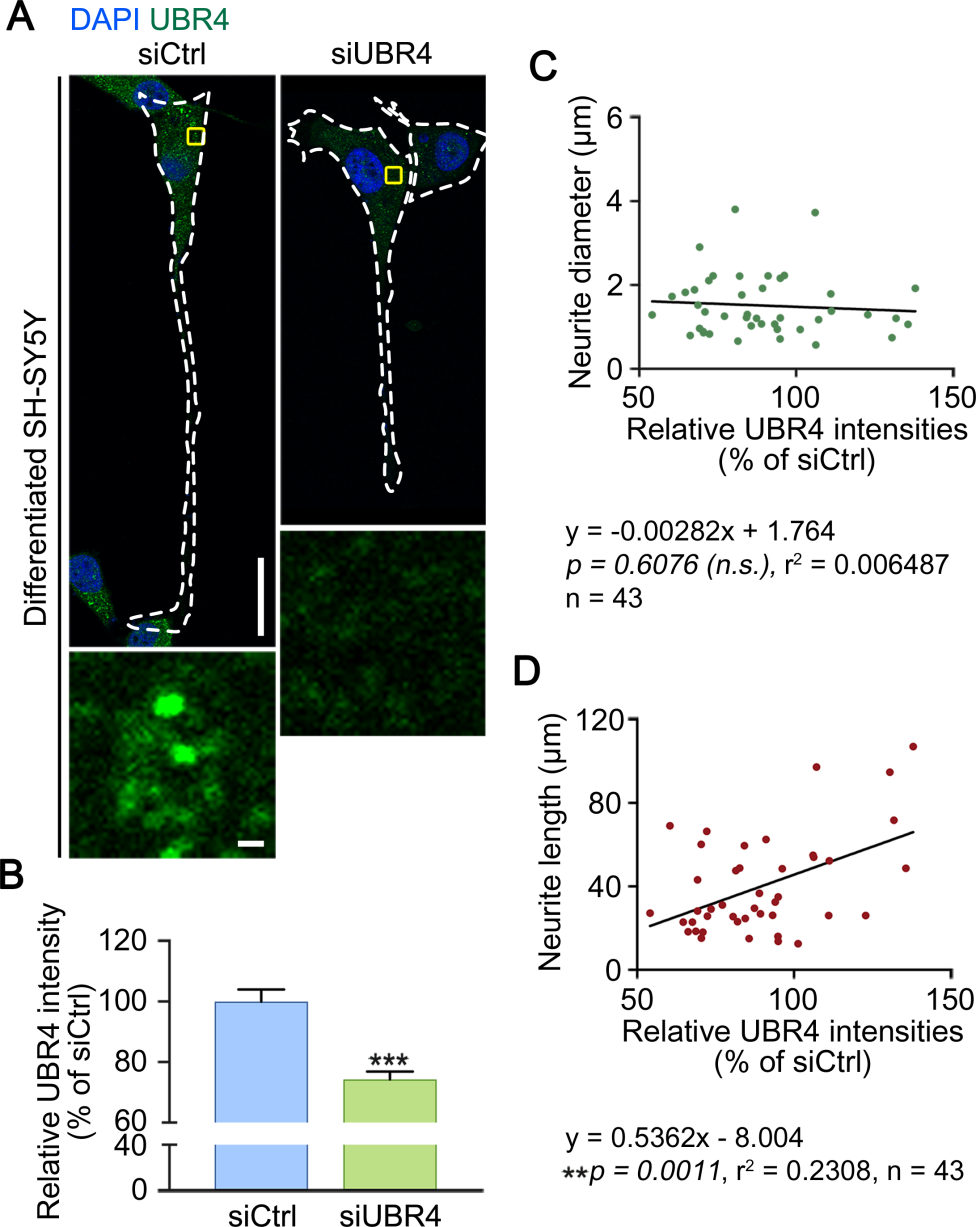
UBR4 depletion causes neurite shortening in differentiated SH-SY5Y cells. (**A**) Immunostaining analyses of UBR4 in differentiated SH-SY5Y cells treated with siCtrl (siControl)- and *siUBR4*. SH-SY5Y cells were differentiated by retinoic acid treatment for 5 days, followed by siRNA treatment for 48 h. White dotted lines delineate the cell boundary. Enlarged views show the areas indicated by yellow rectangles (scale bar = 30 μm). (**B**) Quantification of **A**. Total 25 control and 18 UBR4 knockdown SH-SY5Y cells were observed. ***p < 0.*0001*. Data are presented as mean ± SEM. Statistical significance was determined using unpaired t-test. (**C**) Average neurite diameters (in μm) of control and UBR4 knockdown SH-SY5Y cells. Linear regression (solid line) was applied to this data. The slope indicates that the immunofluorescence intensity of UBR has no relationship with the neurite diameter. n.s., not significant. (**D**) Average neurite length (in μm) of control and UBR4 knockdown SH-SY5Y cells. Linear regression (solid line) was applied to this data. The slope indicates that the immunofluorescence intensity of UBR4 had a correlation with neurite length.

To determine the cell-autonomous function of UBR4 in neuronal cells, human neuroblastoma-derived SH-SY5Y cells were differentiated into the neuronal lineage by treating retinoic acid for 5 days. Immunostaining analyses showed that UBR4 was expressed in neuronal cells as cytosolic puncta throughout the cell body as well as the axon (Fig 3A). Next, we silenced UBR4 in differentiated SH-SY5Y cells and examined their axonal growth. Immunostaining and immunoblotting analyses confirmed that UBR4 was properly knocked down (Fig 3A and 3B). Microscopic inspection revealed that UBR4-knockdown acutely induced the recession of axons as determined by axonal length without any changes in neurite diameter (Fig 3C and 3D). These results suggest that UBR4 is required to maintain axonal integrity of neuronal cells.

### UBR4 plays a role in cell adhesion

**Fig 4:**
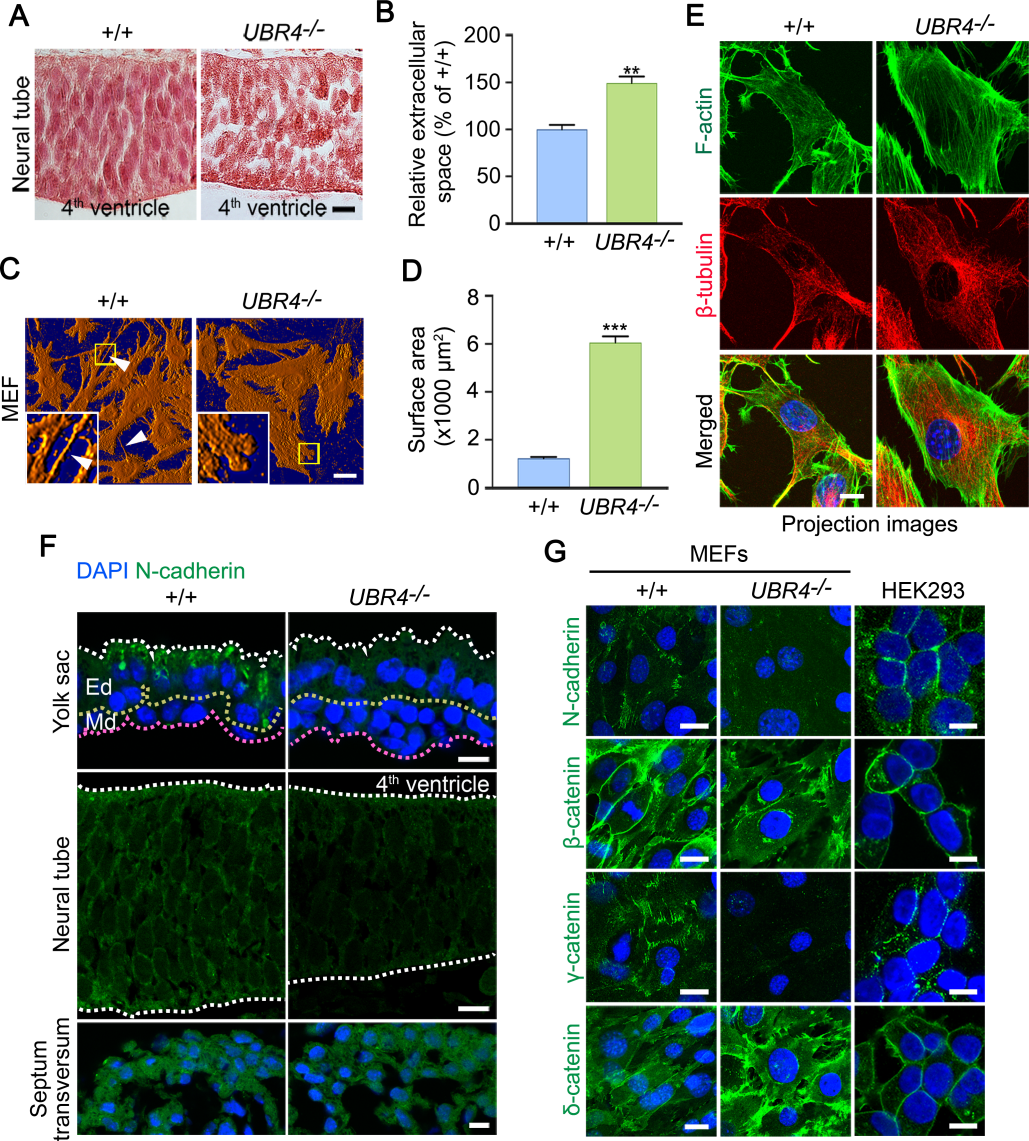
*UBR4*^*-/-*^ embryos are impaired in neurogenesis and cell adhesion. (**A**) H&E staining images of the neural tube of +/+ and *UBR4*^*-/-*^ mouse embryos at E9.5 (scale bar = 10 μm). (**B**) Quantification of the extracellular space between neuronal cells in E9.5 +/+ and *UBR4*^*-/-*^ neural tubes (% of +/+). Extracellular space of *UBR4*^*-/-*^ is larger than that of +/+ (149 ± 7% of +/+, **p = 0.*0040*, n = 4 and n = 8 for +/+ and *UBR4*^*-/-*^ embryos, respectively). Data are presented as mean ± SEM. Statistical significance was determined using two-tailed Mann-Whitney test for non-parametric test. (**C**) The long-term deficiency of UBR4 caused morphological transformation in MEFs. *UBR4*^*-/-*^ MEFs show an increased surface area and lack extended lamellipodia and filopodia. White arrowheads mark extended filopodia (scale bar = 20 μm). Insets: magnified image of area enclosed by yellow lined boxes. (**D**) Quantification of cellular surface (x1000 μm^2^). ***p < 0.*0001* (n = 201 and n = 212 for +/+ and *UBR4*^*-/-*^ MEF, respectively). Data are presented as mean area ± SEM. Statistical significance was determined using two-tailed Mann-Whitney test for non-parametric test. (**E**) Immunostaining analysis of F-actin and β-tubulin on +/+ and *UBR4*^*-/-*^ MEFs. All images are displayed as z-stack maximum projections (scale bar = 10 μm). (**F**) Immunostaining of N-cadherin on cross-sections of +/+ and *UBR4*^*−/−*^ YS and embryos at E9.5. Dotted line represents two distinct layers of the visceral YS. An outer layer derived from the visceral endoderm and an underlying layer derived from the mesoderm. Endodermal layer; Ed, mesodermal layer; Md. White dotted line on neural tube represents outlines of the neural tube (scale bar = 10 μm). (**G**) Immunostaining of cell-cell adjunction proteins on +/+, *UBR4*^*−/−*^ MEFs and HEK293 cell (scale bar = 20 μm for MEFs and 10 μm for HEK293).

In *UBR4*^*-/-*^ embryos at E9.5, migratory mesenchymal cells were more separated from each other as compared with +/+ embryos (Fig 2A). Although to a less degree, the extracellular space of various cells, including neuronal cells of the neural tube, was also invariably expanded than +/+ embryos (Fig 4A and 4B). Quantitative analyses showed that the extracellular space of neural tube in *UBR4*^*-/-*^ embryos were about 1.5-fold (**p = 0.*004)* larger than that of +/+ embryos (Fig 4B). In addition, the cells in *UBR4*^*-/-*^ embryos exhibited morphological abnormalities. Specifically, in +/+ embryos, mesenchymal cells were in a fibrillar shape toward the direction of migration and paracrine signaling. In sharp contrast, the same kinds of cells in *UBR4*^*-/-*^ embryos lost fibrillar shapes and remained arrested as globular shapes (Figs 2A and 4A). These cellular defects were similarly observed in *UBR4*^*-/-*^ MEFs which were morphologically different from +/+ fibroblasts, lacking elongated lamellipodia and filopodia (Fig 4C, arrowheads). Instead, the cytosolic surface areas of *UBR4*^*-/-*^ MEFs were about 5-fold larger than +/+ MEFs (Fig 4D). Despite the increased surface area, *UBR4*^*-/-*^ MEFs retained the cytoskeleton structures as determined by immunostaining analyses of actin filaments and microtubules (Fig 4E). Overall, these results suggest that UBR4 may be required for cell-to-cell adhesion.

We therefore asked whether UBR4 plays a role in cell adhesion by immunostaining N-cadherin, a type-1 transmembrane protein involved in adhesion [50, 51]. The cytoplasmic domain of N-cadherin linked to actin cytoskeleton filaments through interaction with delta-catenin, beta-catenin and gamma-catenin [47]. Immunostaining analyses of +/+ embryos revealed N-cadherin stainings as thin lines along the plasma membrane (Fig 4F). Such expression pattern was markedly downregulated and disorganized in *UBR4*^*-/-*^ embryos (Fig 4F). Notably, N-cadherin signals were more strongly disorganized in the YS and neural tubes that exhibited severe null phenotypes. By contrast, the staining appeared normal in tissues that exhibited relatively mild null phenotypes, such as mesenchymal cells of the septum transversum which gives rise to the thoracic diaphragm and the ventral mesentery of the foregut [52]. Given the results with N-cadherin, we next examined the expression of other cell adhesion proteins such as beta-catenin, delta-catenin, and gamma-catenin in +/+ and *UBR4*^*-/-*^ MEFs. Immunostaining analyses showed that the expression patterns of all these plasma membrane-associated proteins were down-regulated or otherwise disorganized in *UBR4*^*-/-*^ MEFs in a fashion similar to that of N-cadherin (Fig 4G). These results suggest that cell adhesion proteins are not properly presented in the plasma membrane, contributing to pleiotropic developmental defects observed in *UBR4*^*-/-*^ embryos.

### UBR4-loss leads to the depletion of proteins from the plasma membrane

**Fig 5:**
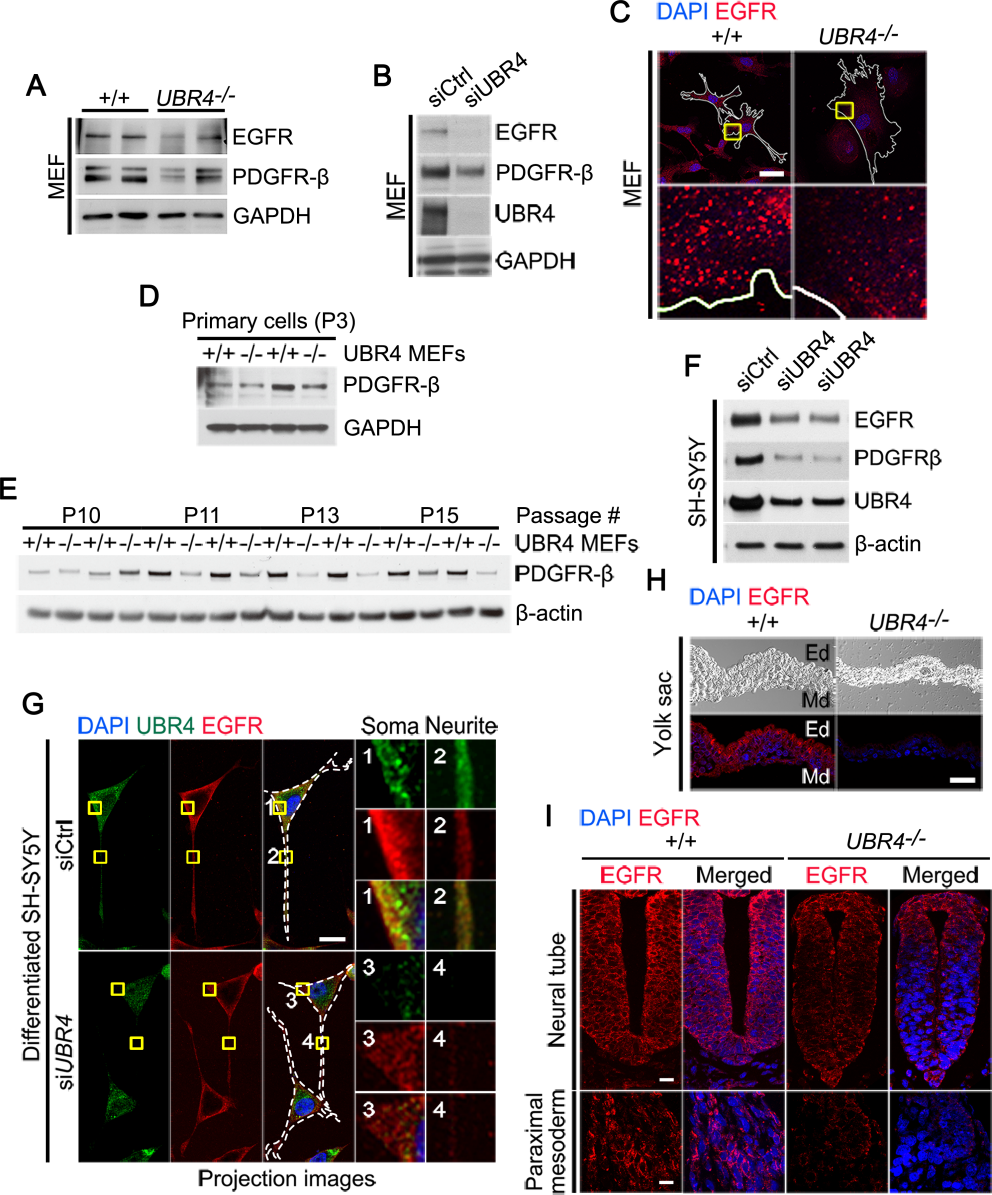
UBR4-loss induces the depletion of EGFR and PDGFR from the plasma membrane. (**A**) Immunoblotting analysis of EGFR and PDGFR-β in *+/+* and *UBR4*^*-/-*^ MEFs. (**B**) Immunoblotting analysis of EGFR and PDGFR-β in siControl (siCtrl) and si*UBR4*-treated MEFs. (**C**) Immunostaining analysis of EGFR using +/+ and *UBR4*^*-/-*^ MEFs. Solid white lines delineate the cell boundary (scale bar = 40 μm). Enlarged views show the areas indicated by yellow rectangles (scale bar = 3 μm). (**D**) Immunoblotting analysis of PDGFR-β using primary cultures of +/+ and *UBR4*^*-/-*^ MEFs at passages 3. GAPDH was probed for loading control. (**E**) Immunoblotting analysis of PDGFR-β in +/+ and *UBR4*^*-/-*^ MEFs at passages 10, 11, 13, and 15. β-Actin was probed for loading control. (**F**) Immunoblotting analysis of EGFR, PDGFR-β, and UBR4 in siControl (siCtrl) and si*UBR4*-treated SH-SY5Y cells. (**G**) Immunostaining of EGFR and UBR4 in siControl (siCtrl) and si*UBR4*-treated differentiated SH-SY5Y. Retinoic acid-derived differentiated SH-SY5Y cells were transiently transfected with siRNA for 48 h. Total 12 images were collected using the confocal microscope at 0.36 μm intervals to create a stack in the Z axis. The fluorescence intensity of EGFR was significantly down-regulated in UBR4 knockdown SH-SY5Y cells. White dotted lines delineate the cell boundary (scale bar = 20 μm). Enlarged views show the areas indicated by yellow rectangles and numbers. (**H**) Immunostaining analysis of EGFR on the cross sections of *+/+* and *UBR4*^*-/-*^ YSs at E9.5. EGFR was significantly down-regulated in the YS of *UBR4*^*-/-*^ embryos at E9.5 (scale bar = 10 μm). Ed, endodermal layer; Md, mesodermal layer. (**I**) Immunostaining analysis of EGFR on the cross-sections of +/+ and *UBR4*^*-/-*^ embryos at E9.5. EGFR was markedly down-regulated in various embryonic tissues, such as the neural tube, and paraximal mesoderm (scale bar = 5 μm).

Cell adhesion proteins assist growth factor receptors like EGFR (epidermal growth factor receptor) and PDGFR (platelet-derived growth factor receptor) and arrange receptor recycling and signaling [53–55]. To assess the impact of UBR4 on the homeostasis of EGFR and PDGFR, we examined their levels in +/+ and *UBR4*^*-/-*^ MEFs. Immunoblotting analysis showed that *UBR4*^*-/-*^ MEFs contained a markedly reduced amout of EGFR (Fig 5A). UBR4 knockdown also acutely induced the depletion of EGFR in MEFs (Fig 5B). When visualized by immunostaining analyses, EGFR^+^ puncta in *UBR4*^*-/-*^ MEFs appeared to be abnormal in size, morphology, and intracellular distribution as compared with those in +/+ MEFs (Fig 5C). Consistent with the results with EGFR, PDGFR-β was also depleted not only in *UBR4*^*-/-*^ MEFs relative to +/+ MEFs (Fig 5A) but also by transient knockdown of UBR4 in MEFs (Fig 5B). As an alternative assay, we established primary MEFs from E9.5 +/+ and *UBR4*^*-/-*^ embryos and monitored PDGFR-β at different passages (Fig 5D and 5E). Although there were variations, we observed a tendency that *UBR4*^*-/-*^ MEF gradually lost PDGFR-β in the course of passaging (Fig 5E). These results suggest that UBR4 is required for the homeostasis of at least some plasma membrane-associated receptors, such as EGFR and PDGFR-β.

To validate these results in the neuronal lineage, we monitored EGFR and PDGFR-β in SH-SY5Y cells (Fig 5F). UBR4 knockdown induced a rapid depletion of EGFR and PDGFR-β in SH-SY5Y cells (Fig 5F). To further characterize the turnover of EGFR in differentiated neurons, we induced the axonal growth of SH-SY5Y cells by treating retinoic acid for 5 days. When visualized using immunostaining analyses, UBR4 knockdown significantly dampened EGFR stainings (Fig 5G). Finally, to extend these findings to animal tissues, we immunostained EGFR on the cross sections of *+/+* and *UBR4*^*-/-*^ mouse YS and embryos at E9.5 (Fig 5H and 5I). The EGFR staining was markedly diminished in *UBR4*^*-/-*^ embryonic tissues, such as the neural tube, paraximal mesoderm, and YS (Fig 5H and 5I). These results suggest that UBR4 has a general role in the homeostasis of at least some plasma membrane-associated receptors.

To assess the impact of PDGFR-β down-regulation on celluar functions, we treated +/+ and *UBR4*^*-/-*^ MEFs with the ligand PDGF-BB and monitored the activation of its downstream signaling pathways (S1 Fig). *UBR4*^*-/-*^ MEFs failed to induce the phosphorylation of PDGFR-β, leading to the dampening of PDGFR-β-dependent downstream signaling pathways as judged by the phosphorylation of src homology 2-containing phosphotyrosine phosphatase (SHP2), serine/threonine kinase AKT, and extracellular signal-regulated kinase 1/2 (ERK1/2) (S1 Fig). These results collectively suggest that UBR4 loss impacts intracelluar signaling pathways.

**Fig 6:**
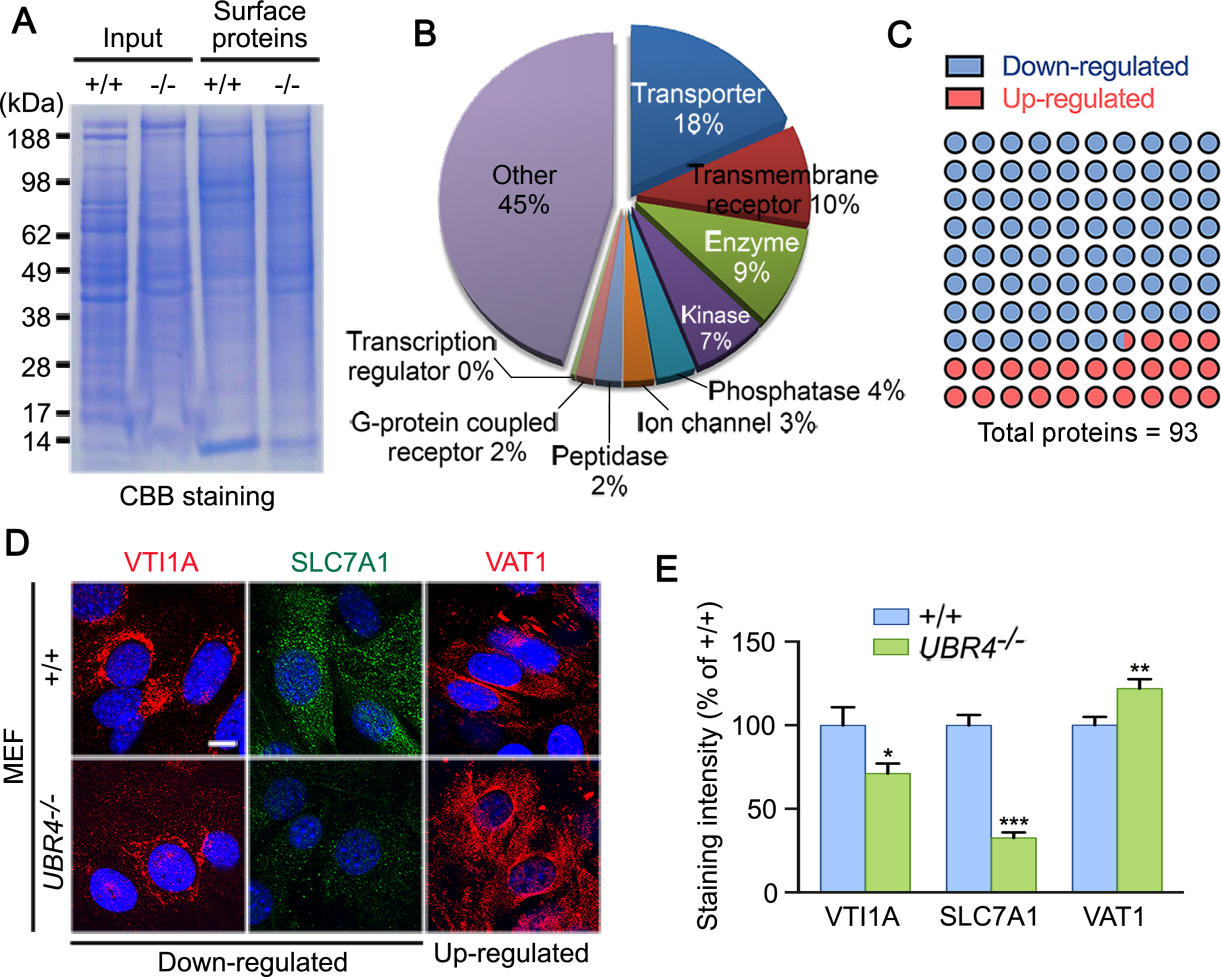
Proteomic analysis of cell surface proteins. (**A**) Coomassie brilliant blue (CBB) staining of purified surface proteins separated using SDS-PAGE. (**B**) Molecular functions of identified cell surface proteins by the Ingenuity Pathway Analysis (IPA) tool. (**C**) Schematic illustration of mass spectrometric data of 93 membrane proteins whose abundance significantly altered in *UBR4*^*-/-*^ MEFs as compared with +/+ MEFs. **(D**) Immunostaining analyses of VTI1A, SLC7A1 and VAT1 (scale bar = 10 μm). (**E**) Quantification of **D**, representing the relative fluorescence intensities for VTI1A, SLC7A1, and VAT1. *p = 0.*0325* vs VTI1A of +/+ (*n* = 13–14 in each group), ***p < 0.*0001* vs SLC7A1 of +/+ (*n* = 13–23 in each group), and **p = 0.*0070* vs VAT1 of +/+ (*n* = 16–22 in each group). Data are presented as mean percentage ± SEM of +/+. Statistical significance was determined using unpaired t-test.

To further characterize the role of UBR4 in the global turnover of plasma membrane-associated proteins, we labeled *in situ* plasma membrane proteins of +/+ and *UBR4*^*-/-*^ MEFs with sulfo-NHS-SS-biotin and partially purified biotinylated proteins using streptavidin Sepharose column. The purified proteins were separated using SDS-PAGE (Fig 6A), followed by in-gel trypsinization and LC-MS/MS analysis. The SEQUEST-SORCERER database search platform identified a total of 1,024 proteins, of which 253 were plasma membrane-associated proteins. Ingenuity Pathway Analysis and PANTHER classification system was used to annotate the function of these identified surface proteins (Fig 6B). Amongst the 253 proteins, 71 were significantly down-regulated in *UBR4*^*-/-*^ MEFs compared to +/+ MEFs (*p* ≤ 0.01) (S1 Table; Fig 6C), and 22 were up-regulated (S2 Table; Fig 6C). To validate the results from mass spectrometry, we performed immunostaining analyses of a few radomly selected hits, including VTI1A, SLC7A1, and VAT1 (Fig 6D). Consistent with the data from mass spectrometric analyses, *UBR4*^*-/-*^ MEFs contained the reduced levels of VTI1A and SLC7A1 and the increased level of VAT1 (Fig 6D and 6E). Overall, these results suggest that UBR4 has a general role in the homeostasis of cell surface proteins.

### UBR4-deficient cells fail to generate MVBs

**Fig 7:**
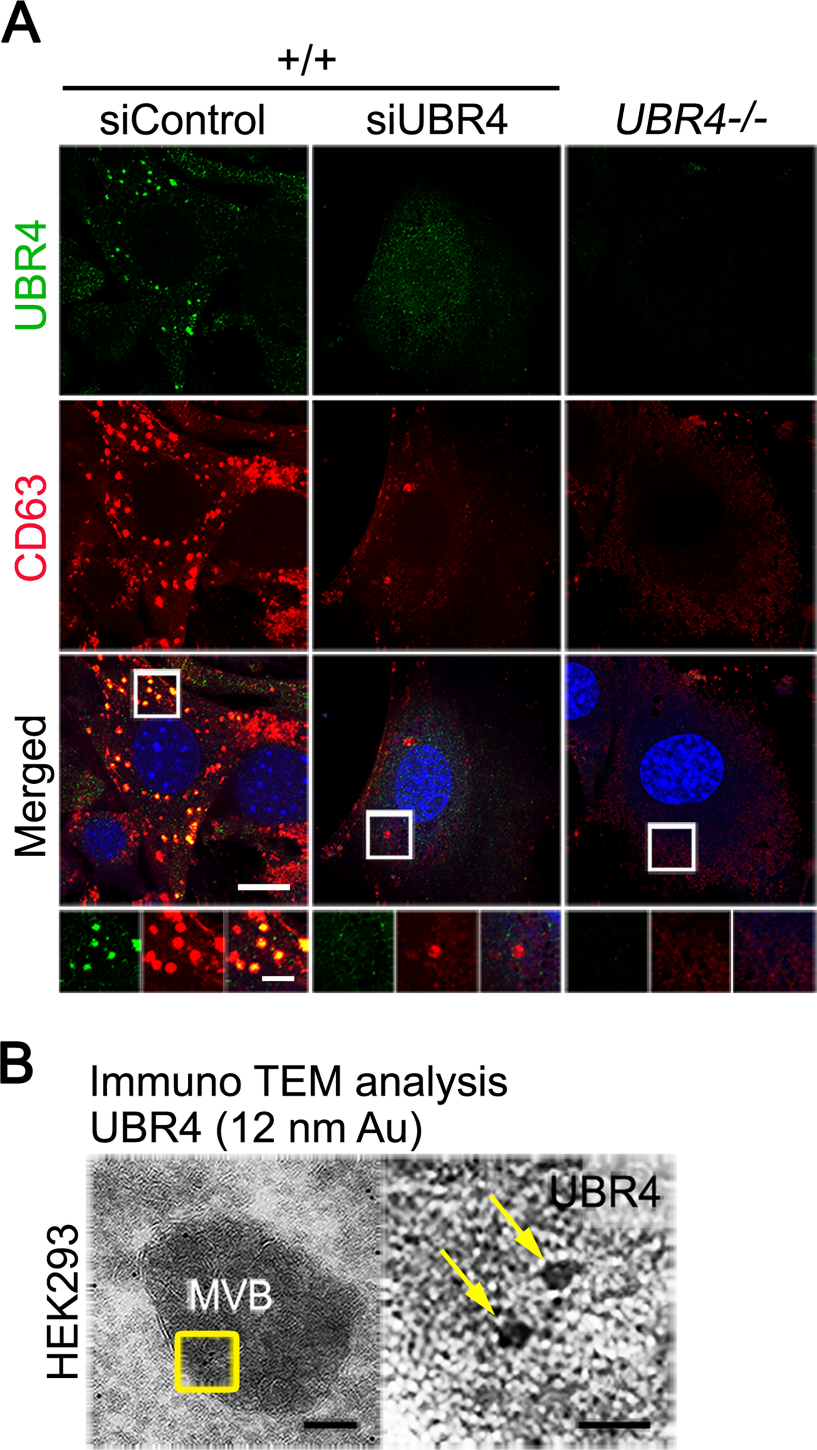
UBR4 is associated with MVBs and required for the formation of MVBs. (**A**) Immunostaining of UBR4 and the MVB marker CD63 on siControl- and *siUBR4*-treated +/+ and *UBR4*^*−/−*^ MEFs (scale bar = 15 μm and 300 nm, respectively). (**B**) Transmission electron microscopy (TEM) of HEK293 cells stably expressing UBR4-V5. Fixed cells were incubated with primary antibody against V5 followed by secondary IgG antibody labelled with 12 nm gold beads. Arrows indicate UBR4-V5 molecules located at an MVB (scale bar = 100 nm for left panel and 20 nm for right panel).

A portion of cell surface proteins and various extracellular materials are endocytosed and degraded through the endosome-lysosome pathway [56]. During this process, cargoes carried in endosomes are deposited to MVBs, which in turn are fused with autophagosomes to form amphisomes, leading to lysosomal degradation [56, 57]. We therefore monitored the formation of MVBs in *+/+* and *UBR4*^*-/-*^ MEFs. Immunostaining analyses of the MVB marker CD63 showed that MVBs were enriched in peri-nuclear regions of +/+ MEFs. By sharp contrast, *UBR4*^*-/-*^ MEFs contained a drastically reduced level of CD63^+^ MVBs (Fig 7A). The down-regulation of MVBs was validated by transient silencing of UBR4 with siRNA (Fig 7A). Consistent with a previous report [32], transmission electron microscopy showed that immunogold labeled UBR4 molecules were deposited to MVBs in HEK293 cells stably expressing UBR4-V5 (Fig 7B), indicating that UBR4 is degraded along the endosome-lysosome pathway. These results suggest that UBR4 is required for the formation of MVBs and proteolysis therein, at least in part providing a mechanism underlying the depletion of cell surface proteins in the absence of UBR4.

## Discussion

We have previously identified UBR4 as an N-recognin of the N-end rule pathway which binds type-1 and type-2 N-degrons of short-lived proteins to facilitate their ubiquitination and proteasomal degradation [6]. In this study, we characterized the null phenotypes of UBR4-deficient mice. Our results show that UBR4-deficient embryos die associated with developmental defects in various processes such as neurogenesis and cardiovascular development (Fig 1). The mutant cells in various tissues do not exhibit tissue-specific morphology as if they cannot receive properly external paracrine signals (Figs 2A and 4A). The mutant cells are more separated from each other, which is in part attributed to the inability to maintain cell surface proteins and, thus, cell adhesion and integrity (Figs 4 and 5). Although the mechanism underlying the role of UBR4 in the homeostasis of cell surface proteins should be further investigated, one such mechanism may be relevant to our observation that the MVBs are not properly generated in the absence of UBR4 (Fig 7).

The N-end rule pathway is a proteolytic system that mediates the degradation of proteins via either the UPS or autophagy [2, 8]. In this pathway, specific N-terminal residues are recognized and directly bound by N-recognins, leading to proteolysis by the proteasome or lysosome. Studies have shown that the RING E3 ligases UBR1 and UBR2 have the UBR box, a 70-residue zinc finger motif, that recognize the N-degrons of various short-lived and misfolded proteins in the cytosol and nucleus [12]. Likewise, the autophagic receptor p62 uses its ZZ domain, a structural homolog of UBR boxes, to recognize N-terminally arginylated proteins from the ER and cytosol to autophagosomes, resulting in proteolysis by lysosomal hydrolases [8, 28]. Overall, these N-recognins bind N-terminal degrons through their conserved substrate recognition domains such as UBR boxes and ZZ domain. It is therefore reasonable to speculate that the functions of UBR4 involve the ability of its UBR box to bind N-degrons. Given the null phenotypes in cell adhesion and the homeostasis of cell surface proteins, we suggest that N-degrons such as the N-terminal Arg residue of arginylated proteins bind the UBR box of UBR4 to modulate the function of UBR4 in these processes, a conjecture to be tested.

Our results show that UBR4-deficient mouse embryos suffer from multiple abnormalities in neurogenesis and other developmental processes. Histological and cytological examinations suggest that the cells in mutant embryos fail to maintain their morphology and adhesion, leading to failure to communicate with each other and advance into more differentiated stages (Figs 1 and 4). Consistently, the expression pattern of cell adhesion markers such as N-cadherin is significantly disorganized (Fig 4F and 4G). Indeed, extensive studies have shown that neurogenesis in embryos is particularly sensitive to misregulation of intercellular adhesion molecules such as immunoglobulins, integrins, cadherins [58, 59]. Why are cell adhesion molecules down-regulated and disorganized in the absence of UBR4? We show that UBR4-loss results in the rapid depletion of other cells surface proteins as well, including EGFR and PDGFR (Fig 5). Moreover, mass spectrometric analyses of purified biotin-conjugated cell surface proteins indicate that a significant portion, although not all, of proteins are depleted from the plasma membrane (Fig 6). These results collectively suggest that UBR4 is required for the turnover of plasma membrane-associated proteins. It is known that cell surface proteins are under constitutive flux into the endosome-lysosome pathway, in which endocytosed membrane proteins are recycled or degraded through endosomal pathways toward lysosomes [56, 57]. Of note, we have recently found that UBR4 is associated with early endosomes through its interaction with calmodulin, degraded by endosomal pathways, and required for the turnover of cell surface proteins (unpublished data). It is therefore reasonable to speculate that the homeostasis of cell surface proteins in UBR4-deficient cells are in part caused by misregulation in their endosome-mediated turnover. We provide evidence that UBR4-deficient cells fail to generate the MVBs in which endosome-cargo complexes are temporarily stored before degradation by lysosomes (Fig 7). Further mechanistic details are to be investigated.

## Materials and methods

### Chemicals and antibodies

Rabbit polyclonal anti-human UBR4 antibody against residues 3755–4160 (1:400) was used for immunohistochemical analysis of YS and embryos [30]. Rabbit polyclonal anti-UBR4 antibody (Bethyl Laboratories, IHC-00640, 1:300) was used for immunostaining of cultured cells. Other antibodies are as follows: rat monoclonal anti-CD63 (R&D, MAB5417, 1:200), mouse monoclonal anti-EGFR (Santa Cruz, SC-374607, 1:200), rabbit monoclonal anti-PDGFR-β (Cell Signaling, 3169, 1:100), mouse monoclonal anti-VTI1A (BD Biosciences, 611220, 1:200), Alexa Fluor 488-conjugated phalloidin (Invitrogen, A12379, 1:100), rabbit polyclonal anti-SLC7A1 (Proteintech, 14195-1-AP, 1:100), mouse monoclonal anti-tubulin (Millipore, 05–661, 1:600), goat polyclonal anti-VAT1 (Santa Cruz, SC-107348, 1:100). For immunoblotting analysis, mouse monoclonal anti-β actin (Sigma, A1978, 1:10,000), rabbit polyclonal anti-UBR4 (Abcam, AB86738, 1:100), and mouse monoclonal anti-GAPDH (Sigma, G8795, 1:20,000) were used. The following secondary antibodies are from Invitrogen: Alexa Fluor 488 goat anti-rabbit IgG (A11034, 1:200), and Alexa Fluor 555 goat anti-mouse IgG (A21424, 1:200). Normal goat serum (ab7481) was obtained from Abcam. Hoechst 33342 (H21492) was obtained from Invitrogen. 4′,6-Diamidino-2-phenylindole dihydrochloride (DAPI; D8417) was obtained from Sigma. Vectashield antifade mounting medium (H1000) was from Vector lab. All other chemicals were reagent grades from Sigma or Merck.

### UBR4 knockout mice

We have previously constructed UBR4-knockout mice, in which the UBR box, a substrate recognition domain for destabilizing N-terminal residues, was replaced with IRES (internal ribosome entry site)-translated tau-lacZ [32]. Animal studies were conducted according to the Guide for the Care and Use of Laboratory Animals published by the protocols (SNU130604-2-10) approved by the Institutional Animal Care and Use Committee at Seoul National University.

### Cell culture

Primary mouse embryonic fibroblasts (MEFs) were established from +/+ and *UBR4*^*-/-*^ embryos at E8.5. The embryos were minced by pipetting in Iscove’s Modified Dulbecco’s Medium (IMDM; Gibco, 31980–022), 15% fetal bovine serum (FBS; Hyclone), 0.1 mM non-essential amino acids (Invitrogen), 0.1 mM β-mercaptoethanol, and 100 U/ml penicillin/streptomycin (Gibco, 15140–148). The cells were seeded on the gelatinized 35 mm culture dish. Immortalized cell lines were established from primary MEFs through crisis-mediated immortalization over 10 passages [32]. CCL2 HeLa and HEK293 cell were purchased from the American Type Culture Collection (ATCC). SH-SY5Y cells were provided by Prof. Onyou Hwang (University of Ulsan College of Medicine, Seoul, Republic of Korea). All the cell lines were determined to be negative in a mycoplasma test using a MycoAlert detection kit (Lonza, LT07-118). HeLa and HEK293 cell were cultured in Dulbecco’s Modified Eagle’s Medium (DMEM; Gibco,10566016) and SH-SY5Y cells were cultured in DMEM/F-12 (Gibco, 10565–042). The media was supplemented with 10% FBS and 100 units/mL penicillin/streptomycin. All the culture plates and the cell lines were maintained at 37°C and 5% CO_2_ in a humidified incubator.

### RNA interference assay

Reagents for siRNA silencing were purchased from Life Technologies. Transfection was performed according to the manufacture’s protocol. Briefly, HEK293 and SH-SY5Y cells were transfected with either siControl (cat. #4390843) or UBR4 siRNA (cat. #4392420, ID #23628) at a final concentration of 10 nM using Lipofectamine RNAiMAX reagent (Invitrogen, 13778150). Approximately 48 hours after siRNA silencing, cells were harvested for immunoblotting and immunocytochemical analyses. The sequences of *UBR4* siRNAs are 5’-GCCUGUUCGAAAGCGCAAA (sense) and 5’-UUUGCGCUUUCGAACAGGC (antisense).

### Immunoblotting analysis

Cells were washed with cold phosphate buffered saline (PBS) and lysed using RIPA buffer (50 mM Tris-HCl, 150 mM NaCl, 1% NP-40, 1% sodium deoxycholate, and 0.1% SDS) containing freshly prepared protease inhibitor cocktail (Sigma, P8340). Lysates were centrifuged at 14,000 rpm for 20 min at 4°C, and the supernatants were used for immunoblotting. Protein concentrations were measured using the BCA protein assay kit (Pierce, 23225). The samples were diluted with 2X Laemmli sample buffer (65.8 mM Tris-HCl, pH 6.8, 26.3% (w/v) glycerol, 2.1% SDS, 0.01% bromophenol blue, Bio-Rad, 161–0737) or in Lithium dodecyl sulfate (LDS) sample buffer (Invitrogen, NP0007) with a reducing reagent, followed by heating for 10 min at 70°C. Whole cell lysates were separated by sodium dodecyl sulfate (SDS)-polyacrylamide gel electrophoresis and transferred to polyvinylidene difluoride membranes (Millipore, IPVH00010). Blocking was done using TBS-T (20 mM Tris-HCl, pH 7.5, 150 mM NaCl, and 0.05% (v/v) Tween 20) containing 1% BSA for 1 h at room temperature, and the membrane was incubated with antibodies diluted with the blocking solution for overnight at 4°C.

### Histology and immunohistochemistry

For histological analysis, embryos were fixed overnight at 4°C in 4% paraformaldehyde (PFA) in PBS, pH 7.4. Fixed embryos were gradually dehydrated with 70%, 90%, and 100% EtOH, followed by immersion in Neo-Clear (Millipore, 65351). Tissues were embedded in paraffin wax at 58°C and sectioned transversely or sagittally with 7 μm thickness. Immunostaining of paraffin sections and whole-mount immunohistochemical staining of embryos were performed as described [32]. Paraffin-embedded slides were freshly treated with Neo-Clear twice for 10 min each, followed by gradual rehydration in EtOH (100%, 90%, 80%, and 70%; 6 min each) and water for 20 min. For immunohistochemistry, the slides were treated with blocking solution (5% normal goat serum and 0.2% Triton X-100 in PBS) for 1 h and incubated with primary antibodies and subsequently secondary antibodies. Confocal images were taken with a Zeiss LSM 700 laser scanning confocal microscope equipped with Zeiss C-Apochromat 60x (1.2 NA) and 40x (1.2 NA) water immersion lens and analyzed using ZEN (black edition) 2012 SP5 software (Zeiss). Using the ZEN software, z-stacks of images covering the entire cell thickness were acquired and projected maximally. Image processing and annotation was done with Adobe Photoshop, Adobe Illustrator and Fiji software [60].

### Immunocytochemistry of cultured cells

Three 22mm^2^ coverslips per well were placed in 6-well plates, followed by incubation of diluted poly-L-lysine (1:10 in sterile deionized water; Sigma, P8920) for 30 min at room temperature. After washing once, the plate was exposed under UV radiation lamp overnight in a fume hood. Cells were cultured in the plate for further experiments. Cells were fixed in 4% PFA in PBS, for 30 min at room temperature. After washing twice with PBS, the cells were incubated for 1 h in blocking solution (5% normal goat serum and 0.3% Triton X-100 in 0.1 M PBS). After blocking, immunohistochemical processes were conducted as described above. N-SIM images were taken with a Nikon Eclipse Ti inverted microscope (Nikon, Tokyo, Japan) equipped with a Nikon Apo TIRF 100x (1.49 NA) oil-immersion lens. The SIM images were reconstructed from the raw images through the Nikon NIS-element (ver. 3.22.10.).

### Biotinylation and affinity purification of cell surface proteins

Cells (10^7^) were washed three times with cold PBS and biotinylated for 15 min at room temperature using 0.5 mg/ml sulfo-NHS-SS-biotin (Thermo Fisher, 21331), and then washed with 50 mM Tris-HCl (pH 7.5). Cells were harvested by centrifugation (500 ×*g*, 5 min, 4°C) with a buffer containing 0.5 mM oxidized glutathione (Sigma) in PBS. The cell pellet was resuspended in PBS containing 2% NP-40, 0.2% SDS, 0.1mM oxidized glutathion, and protease inhibitor cocktail (Roche Diagnostics). Cells were then lysed by sonication on ice. Protein concentrations were measured using the Micro BCA Protein Assay Kit (Thermo, 23235) [61, 62]. Protein mixtures were diluted to a concentration of 0.6 mg/mL using PBS and biotinylated proteins were isolated using a Streptavidin Sepharose High Performance column (GE Healthcare) following the manufacturer’s protocol [63]. Purified proteins were precipitated with acetone, resuspended with 0.1% SDS in 100 mM ammonium bicarbonate, and protein concentration was determined by the BCA protein assay.

### SDS-PAGE fractionation and in-gel digestion

Fifty micrograms of protein samples were fractionated on 4–12% Bis-Tris gel (Invitrogen) and stained with Coomassie Brilliant Blue (Sigma). Each gel lane was cut into eight pieces and subjected to in-gel tryptic digestion following the general protocol [64]. Briefly, protein bands were excised, destained, and washed. Proteins were reduced with 20 mM dithiothreitol and alkylated with 55 mM iodoacetamide. After dehydration, the proteins were digested with 12.5 ng/μL sequencing grade modified porcine trypsin (Promega) in 50 mM ammonium bicarbonate overnight at 37°C. Peptides were extracted from the gel pieces with 50% (v/v) acetonitrile in 5% (v/v) formic acid and dried under vacuum.

### Mass spectrometry analysis and relative protein quantification

Peptides were resuspended in Solvent A (0.1% formic acid in water) and loaded onto an in-house packed 75 μm (inner diameter micro-capillary) x 10 cm C_18_ column and separated with a linear gradient of 5–32% Solvent B (0.1% formic acid in acetonitrile) for 70 min at a flow rate 300 nL/min. MS spectra were recorded on Q-Exactive (Thermo Fisher) interfaced with a nano-UPLC system (Easy nLC, Thermo Fisher). The Q-Exactive was operated in data-dependent mode with one survey MS scan at 400–1600 *m/z* followed by ten MS/MS scans by normalized collision energy of 30 eV, and the duration time of dynamic exclusion was 30 s. Collected MS/MS data were converted into mzXML files through the Trans Proteomic pipeline (TPP) (version 4.5) and searched against the decoy IPI Mouse database (version 3.87, 119072 entries) for the estimation of false discovery rate (FDR) with the SEQUEST® (Thermo Fisher Scientific; version v.27, rev. 11) in SORCERER^TM^ (Sage-N Research, version 3.5) search platform. Precursor and fragment ion tolerance were set to 50 ppm and 0.5 Da, respectively. Trypsin was chosen as the enzyme and up to two missed cleavages were allowed. Carbamidomethyl of cysteine was considered as the fixed modification, and methionine oxidation as variable modification. Scaffold software (version 3.4.9, Proteome Software Inc., Portland, OR) was used to validate MS/MS based peptide and protein identifications. Peptide and protein identifications were accepted if they could be established at greater than 95% and 99% probability respectively, as specified by the Peptide and Protein Prophet algorithm, and protein identification contained at least two identified peptides [65, 66]. Relative protein quantitation was accomplished using the spectral counting. The MS/MS data were normalized to compare abundances of proteins between samples using Scaffold software. The normalized spectral counts from duplicate measurements of the +/+ and *UBR4*^*-/-*^ MEFs cells were compared using the R program with PLGEM software (http://www.bioconductor.org) in order to identify statistically significant protein changes between the two cell lines [67]. The subcellular localization of the identified proteins was classified using Ingenuity Pathway Analysis (IPA) (Ingenuity® Systems, www.ingenuity.com).

### Immunogold labelling for transmission electron microscopy

Immunoelectron microscopy experiment was performed as previously described [32]. UBR4-V5 stably expressing HEK293 cells were fixed in 2% PFA in PBS for 1 h, followed by PBS washing. The fixed cells were collected by scraping and resuspended in 3% gelatin. Solidified gelatin on ice was fixed again with 2% PFA for 15 min. After sequential cryoprotections with 2.3 M sucrose and PVP solution overnight, samples were frozen in liquid nitrogen and were trimmed into 0.5 mm cubes, which were sectioned by cryo-microtome (Leica EM Crion) in 70 nm sections. The primary antibody against V5 was diluted in 0.1 M PBS, supplemented with 0.5% bovine serum albumin (BSA), 0.15% glycine. Secondary antibody, 12nm gold beads labelled donkey anti-rabbit IgG (Jackson ImmunoResearch), was diluted to 1: 25. Sections were incubated with 2.5% glutaraldehyde for 10 min and with 2% Neutral UA acetate for 7 min, followed by incubation in 4% uranyl acetate and methyl cellulose for contrasting and drying. After drying, samples were recorded using JEOL JEM1011 TEM with high resolution AMT digital camera (Peabody, MA).

### Statistical analysis

All numerical data are presented as means ± SEM or mean percentages ± SEM. Western blots shown are representative of three or more independent experiments. Comparisons among treatment groups were performed with one-way analysis of variance (ANOVA) and Tukey test or Dunnett’s multiple comparison as a post hoc comparison. Instances involving only two comparisons were evaluated with unpaired t-test or two-tailed Mann-Whitney test for non-parametric test. Statistical significance was accepted if the null hypothesis was rejected with *p < 0*.*05*. All statistical analyses were determined using Prism 7.0c software (GraphPad, La Jolla, CA).

## Supporting information

S1 Fig UBR4 is required for PDGF-PDGFR signaling pathways. (**A**) Immunoblotting analysis of PDGF-BB stimulated +/+ and *UBR4*^*-/-*^ MEFs were probed for PDGFR-β, p-PDGFR-β, p-SHP2, p-AKT, ERK1/2, p-ERK1/2, and EIF4E. Cells were stimulated with 100 ng/ml PDGF-BB for 7.5, 15, 45, 120, and 240 min. (**B**) Immunoblotting analysis of +/+ and *UBR4*^*-/-*^ MEFs treated with PDGF-BB were probed for ERK1/2 and p-ERK1/2. Cells were stimulated with 100 ng/ml PDGF-BB for 0, 7.5, 15, 30, and 60 min.
(TIF)

S1 Table The list of 71 membrane proteins down-regulated in *UBR4*^*-/-*^ MEF cells compared with +/+ MEFs (*p ≤ 0*.*01*). (PDF)

S2 Table The list of 22 membrane proteins up-regulated in *UBR4*^*-/-*^ MEF cells compared with +/+ MEFs (*p ≤ 0*.*01*). (TIFF)
