# Supplementary figures and images for: The N-recognin UBR4 of the N-end rule pathway is required for neurogenesis and homeostasis of cell surface proteins
Source: PLoS One. 2018 Aug 29;13(8):e0202260. doi: 10.1371/journal.pone.0202260 (PMC6114712; doi:10.1371/journal.pone.0202260)

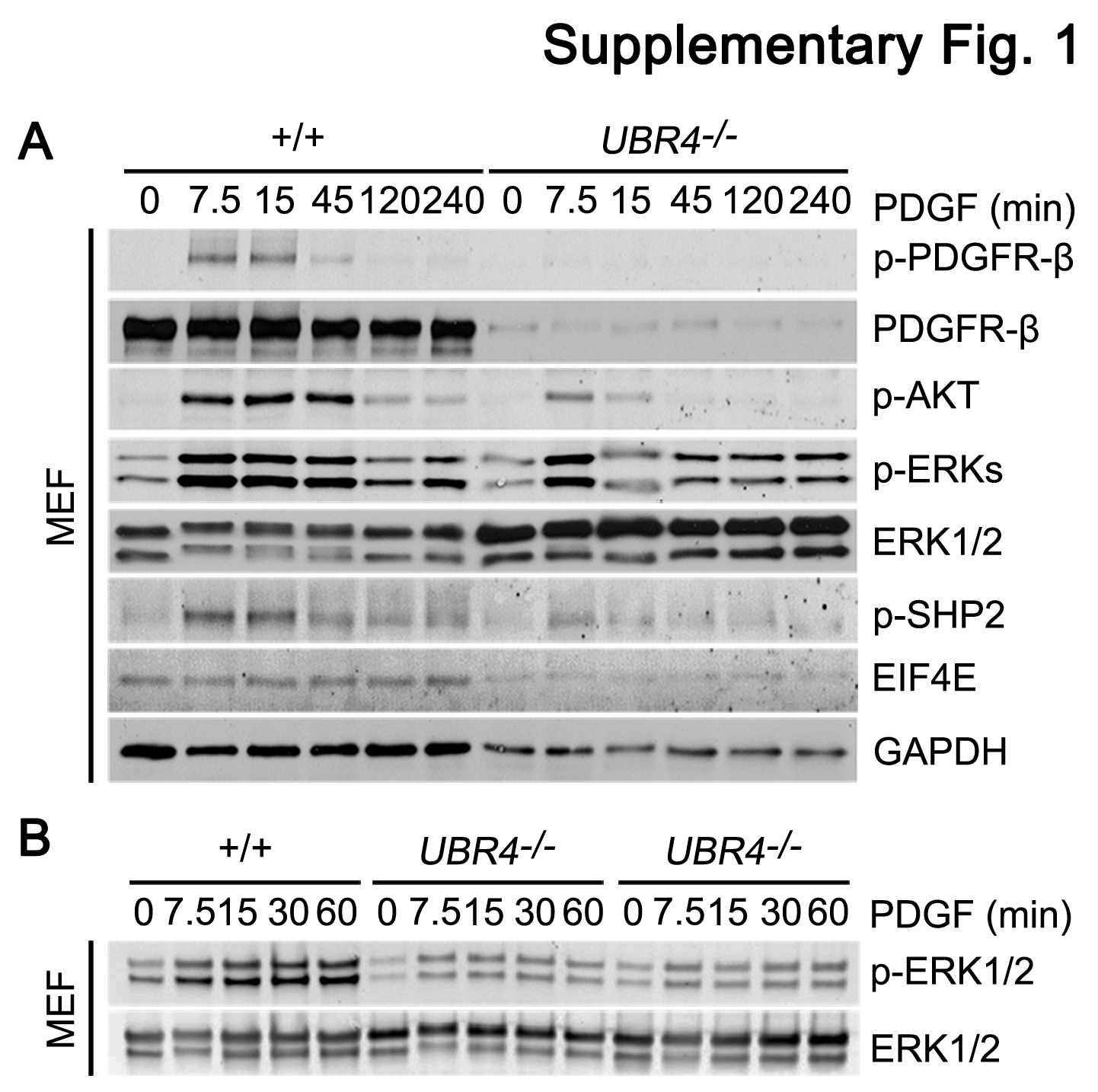

Supplement: S1 Fig — (A) Immunoblotting analysis of PDGF-BB stimulated +/+ and UBR4-/- MEFs were probed for PDGFR-β, p-PDGFR-β, p-SHP2, p-AKT, ERK1/2, p-ERK1/2, and EIF4E. Cells were stimulated with 100 ng/ml PDGF-BB for 7.5, 15, 45, 120, and 240 min. (B) Immunoblotting analysis of +/+ and UBR4-/- MEFs treated with PDGF-BB were probed for ERK1/2 and p-ERK1/2. Cells were stimulated with 100 ng/ml PDGF-BB for 0, 7.5, 15, 30, and 60 min. (TIF) [file pone.0202260.s001.tif]

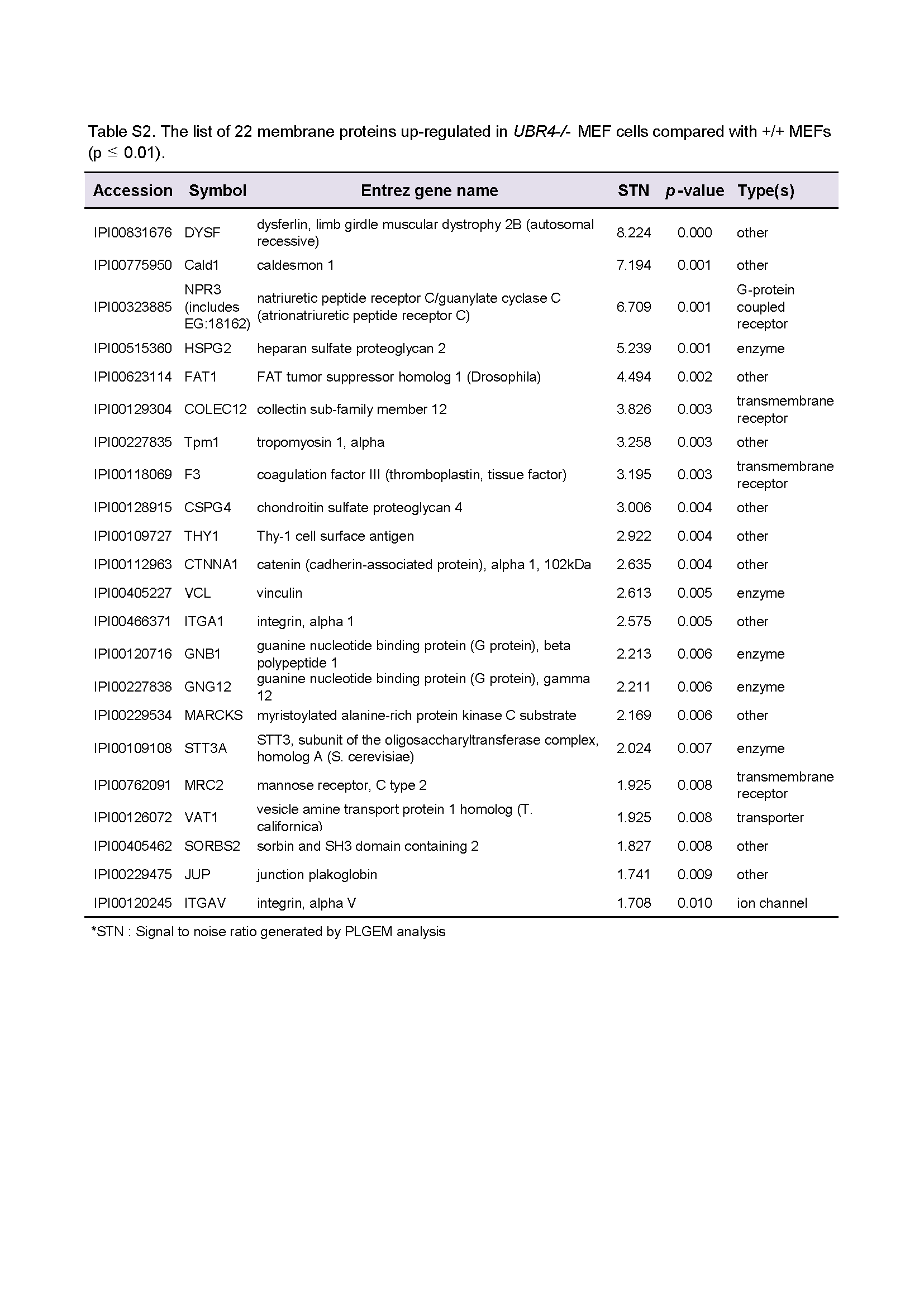

Supplement: S2 Table — (TIFF) [file pone.0202260.s003.tiff]
