# Supplementary material for: The N-recognin UBR4 of the N-end rule pathway is required for neurogenesis and homeostasis of cell surface proteins
Source: PLoS One. 2018 Aug 29;13(8):e0202260. doi: 10.1371/journal.pone.0202260 (PMC6114712; doi:10.1371/journal.pone.0202260)
Supplement: S1 Table — (PDF) [file pone.0202260.s002.pdf]

Table S1. The list of 71 membrane proteins down-regulated in *UBR4-/-* MEF cells compared with +/+ MEFs ($p \leq 0.01$).

Accession	Symbol	Entrez gene name	STN	*p*-value	Type(s)
IPI00468633	SLC6A9	solute carrier family 6 (neurotransmitter transporter, glycine), member 9	-1.626	0.010	transporter
IPI00115117	STOML2	stomatin (EPB72)-like 2	-1.626	0.010	other
IPI00331524	ERLIN1	ER lipid raft associated 1	-1.626	0.010	other
IPI00408489	DSCAML1	Down syndrome cell adhesion molecule like 1	-1.626	0.010	other
IPI00323822	RRAS2	related RAS viral (r-ras) oncogene homolog 2	-1.683	0.010	enzyme
IPI00122971	NCAM1	neural cell adhesion molecule 1	-1.694	0.010	other
IPI00110588	MSN	moesin	-1.727	0.009	other
IPI00648802	EPHB2	EPH receptor B2	-1.727	0.009	kinase
IPI00123040	PTPRK	protein tyrosine phosphatase, receptor type, K	-1.809	0.009	phosphatase
IPI00230395	ANXA1	annexin A1	-1.822	0.008	other
IPI00122273	DAG1	dystroglycan 1 (dystrophin-associated glycoprotein 1)	-1.827	0.008	transmembrane receptor
IPI00131540	VTI1A	vesicle transport through interaction with t-SNAREs homolog 1A (yeast)	-1.827	0.008	transporter
IPI00228617	GNAI2	guanine nucleotide binding protein (G protein), alpha inhibiting activity polypeptide 2	-1.852	0.008	enzyme
IPI00312509	AXL	AXL receptor tyrosine kinase	-1.987	0.007	kinase
IPI00129298	PALM	paralemmin	-1.987	0.007	other
IPI00129220	EPHA2	EPH receptor A2	-2.117	0.006	kinase
IPI00121827	PDGFRB	platelet-derived growth factor receptor, beta polypeptide	-2.190	0.006	kinase
IPI00664442	SLC4A7	solute carrier family 4, sodium bicarbonate cotransporter, member 7	-2.196	0.006	transporter
IPI00313884	PODXL	podocalyxin-like	-2.211	0.006	kinase
IPI00331175	SLC12A7	solute carrier family 12 (potassium/chloride transporters), member 7	-2.211	0.006	transporter
IPI00120930	PLSCR1	phospholipid scramblase 1	-2.211	0.006	enzyme
IPI00270376	CXADR	coxsackie virus and adenovirus receptor	-2.211	0.006	transmembrane receptor
IPI00123996	NRP1 (includes EG:18186)	neuropilin 1	-2.304	0.005	transmembrane receptor
IPI00123183	AQP1	aquaporin 1 (Colton blood group)	-2.304	0.005	transporter
IPI00124221	ATP1B3	ATPase, Na+/K+ transporting, beta 3 polypeptide	-2.366	0.005	transporter
IPI00416751	SLC29A1	solute carrier family 29 (nucleoside transporters), member 1	-2.413	0.005	transporter
IPI00137311	PLXNA1	plexin A1	-2.485	0.005	transmembrane receptor
IPI00405742	PLXNB2	plexin B2	-2.550	0.004	transmembrane receptor
IPI00132474	ITGB1	integrin, beta 1 (fibronectin receptor, beta polypeptide, antigen CD29 includes MDF2, MSK12)	-2.550	0.004	transmembrane receptor
IPI00121634	SLC7A1	solute carrier family 7 (cationic amino acid transporter, y+ system), member 1	-2.559	0.004	transporter
IPI00130648	VLDLR	very low density lipoprotein receptor	-2.575	0.004	transporter

IPI00117181	FLOT1	flotillin 1	-2.589	0.004	other
IPI00118569	GNA13	guanine nucleotide binding protein (G protein), alpha 13	-2.589	0.004	enzyme
IPI00308971	IGF2R	insulin-like growth factor 2 receptor	-2.608	0.004	transmembrane receptor
IPI00330539	KIRREL	kin of IRRE like (Drosophila)	-2.708	0.004	other
IPI00468236	ANO6	anoctamin 6	-2.708	0.004	ion channel
IPI00135130	SLC1A4	solute carrier family 1 (glutamate/neutral amino acid transporter), member 4	-2.725	0.004	transporter
IPI00124830	CD47	CD47 molecule	-2.728	0.004	other
IPI00465786	TLN1	talin 1	-2.756	0.004	other
IPI00754549	FLOT2	flotillin 2	-2.855	0.004	other
IPI00420589	IGSF3	immunoglobulin superfamily, member 3	-3.256	0.003	other
IPI00311682	ATP1A1	ATPase, Na+/K+ transporting, alpha 1 polypeptide	-3.359	0.003	transporter
IPI00129253	LY75	lymphocyte antigen 75	-3.406	0.003	other
IPI00117829	CAV1	caveolin 1, caveolae protein, 22kDa	-3.463	0.003	other
IPI00113798	SNAP23	synaptosomal-associated protein, 23kDa	-3.501	0.003	transporter
IPI00115976	ITGA5	integrin, alpha 5 (fibronectin receptor, alpha polypeptide)	-3.532	0.003	other
IPI00308691	SLC2A1	solute carrier family 2 (facilitated glucose transporter), member 1	-3.657	0.002	transporter
IPI00115546	GNAO1	guanine nucleotide binding protein (G protein), alpha activating activity polypeptide O	-3.657	0.002	enzyme
IPI00223769	CD44	CD44 molecule (Indian blood group)	-3.706	0.002	enzyme
IPI00113869	BSG	basigin (Ok blood group)	-3.720	0.002	transporter
IPI00125832	CAV2	caveolin 2	-3.720	0.002	other
IPI00128152	Abcb1b	ATP-binding cassette, sub-family B (MDR/TAP), member 1B	-3.735	0.002	transporter
IPI00138716	RAP2B	RAP2B, member of RAS oncogene family	-3.735	0.002	enzyme
IPI00875340	SLC1A5	solute carrier family 1 (neutral amino acid transporter), member 5	-4.118	0.002	transporter
IPI00321753	GPRC5A	G protein-coupled receptor, family C, group 5, member A	-4.118	0.002	G-protein coupled receptor
IPI00137194	SLC16A1	solute carrier family 16, member 1 (monocarboxylic acid transporter 1)	-4.434	0.002	transporter
IPI00137336	GPC1	glypican 1	-4.581	0.001	transmembrane receptor
IPI00653515	LDLR	low density lipoprotein receptor	-4.706	0.001	transporter
IPI00556827	ATP2B1	ATPase, Ca++ transporting, plasma membrane 1	-5.103	0.001	transporter
IPI00126090	ITGA3	integrin, alpha 3 (antigen CD49C, alpha 3 subunit of VLA-3 receptor)	-5.111	0.001	other
IPI00463589	ATP2B4	ATPase, Ca++ transporting, plasma membrane 4	-5.336	0.001	transporter
IPI00135324	SLC12A2	solute carrier family 12 (sodium/potassium/chloride transporters), member 2	-5.339	0.001	transporter
IPI00126834	VCAM1	vascular cell adhesion molecule 1	-5.630	0.001	other
IPI00129395	SLC7A5	solute carrier family 7 (amino acid transporter light chain, L system), member 5	-5.943	0.001	transporter
IPI00229703	VAMP2	vesicle-associated membrane protein 2 (synaptobrevin 2)	-6.411	0.001	other

IPI00114641	SLC3A2	solute carrier family 3 (activators of dibasic and neutral amino acid transport), member 2	-7.015	0.001	transporter
IPI00153809	CD109	CD109 molecule	-7.269	0.000	other
IPI00923039	ABCC4 (includes EG:10257)	ATP-binding cassette, sub-family C (CFTR/MRP), member 4	-7.505	0.000	transporter
IPI00129915	ABCC1	ATP-binding cassette, sub-family C (CFTR/MRP), member 1	-8.787	0.000	transporter
IPI00132276	VAMP3	vesicle-associated membrane protein 3 (cellubrevin)	-10.074	0.000	other
IPI00124700	TFRC	transferrin receptor (p90, CD71)	-12.338	0.000	transporter

*STN : Signal to noise ratio generated by PLGEM analysis
